# Calcination of Ca-Based Sorbents in the Presence of Steam for Sorption-Enhanced Gasification Applications

**DOI:** 10.3390/ma19101959

**Published:** 2026-05-09

**Authors:** William A. González, Susanna Nilsson, Diego Fuentes-Cano, Alicia Ronda, Alberto Gómez-Barea

**Affiliations:** 1 Chemical and Environmental Engineering Department, Escuela Técnica Superior de Ingeniería, University of Seville, Camino de los Descubrimientos s/n, 41092 Seville, Spain; wilgonmes@alum.us.es (W.A.G.);; 2 Fundación Ciudad de la Energía, CIUDEN, Centro de Desarrollo de Tecnologías, 24492 Cubillos de Sil, Spain

**Keywords:** calcium looping, calcination, cycling deactivation, kinetics, fluidized bed

## Abstract

The calcination kinetics of limestone and dolomite under conditions relevant to sorption-enhanced gasification (SEG) were investigated: mild temperature (775–850 °C), low CO_2_ partial pressure (0.05–0.10 bar), and a steam-rich (H_2_O balance) atmosphere. Experiments with two Ca-based sorbents (limestone and dolomite) were conducted in a fluidized bed reactor to assess both initial calcination kinetics and multicycle deactivation during 10 cycles under SEG carbonation conditions at 650 °C. Dolomite exhibited markedly higher calcination rates than limestone, which is consistent with the structural modifications induced by MgCO_3_ decomposition and the presence of MgO, resulting in a slightly lower apparent activation energy (115.96 kJ mol^−1^ for dolomite compared to 120.27 kJ mol^−1^ for limestone). Both sorbents showed a strong sensitivity to the deviation from the equilibrium CO_2_ partial pressure, with reaction orders near 2. The presence of steam was confirmed to have a significant catalytic effect, accelerating the first-cycle calcination rate compared to dry N_2_ conditions. Sorbent deactivation caused by sintering was more pronounced at higher temperatures and CO_2_ pressures. Dolomite showed significantly less deactivation, compared to limestone, which can be attributed to the increase in structural stability due to the presence of MgO. The kinetics obtained in this work contribute to the design of stable SEG based on dual fluidized bed reactors, particularly to assist in the selection of calcination operating conditions to minimize sorbent deactivation and in the development of stable CO_2_-sorbents.

## 1. Introduction

The search for sustainable alternatives for hydrogen production, a key energy carrier in the transition toward a defossilized economy, is of vital importance in the current global context. However, hydrogen is currently produced predominantly through the reforming of fossil fuels, emitting a large amount of CO_2_ to the atmosphere. Renewable energy sources such as biomass and solar energy are increasingly being considered as promising alternatives to produce hydrogen in future decarbonized energy systems. Much attention has been devoted to optimizing hydrogen production from steam biomass gasification. The addition of CaO enables in situ CO_2_ capture, lowering its partial pressure and thereby promoting the water–gas shift reaction in the forward direction, which enhances hydrogen yield [1]. As a result, steam gasification has emerged as one of the most promising technologies for sustainable hydrogen production when combined with CaO-based CO_2_ capture. Furthermore, carrying out the gasification in a fluidized bed allows for flexible operation and high efficiency and enables scale-up of the process.

A practical implementation of this concept involves the use of CaO-based sorbents in a gasification/carbonation–calcination cycle, commonly known as calcium looping gasification (CaLG), or sorption-enhanced gasification (SEG). Several configurations have been proposed for the continuous operation of CaLG/SEG systems. The earliest approach, theoretically proposed, involved two parallel fixed-bed reactors operating alternately to achieve continuous processing [2]. However, this configuration has not been considered suitable for large-scale applications. In contrast, the use of dual fluidized bed gasifiers (FBG) has emerged as a more viable option for continuous large-scale operation. FBG systems offer multiple advantages, including high fuel flexibility, uniform solid–gas contact, efficient heat and mass transfer, high conversion efficiency, and ease of integration into industrial settings [3]. These features make dual fluidized bed configurations one of the most widely explored and promising approaches for the development of biomass-based efficient and clean hydrogen production systems [4]. It has been found that a syngas containing approximately 80%vol of H_2_ could theoretically be obtained at temperatures ranging from 500 °C to 700 °C, compared to an H_2_ content between 35%vol and 45%vol without in situ CO_2_ capture [5,6].

Recently, the integration of SEG with renewable heat sources, particularly concentrated solar energy, has gained increasing attention as a pathway to produce renewable hydrogen. In this context, different approaches have been proposed and analyzed, including several solar-driven SEG configurations [7,8,9], where operation under thermally mild calcination conditions is essential to match achievable solar reactor temperatures and enhance sorbent durability. This motivates the present study, which explores the calcination behavior of natural Ca-based sorbents under steam-rich and low CO_2_ environments representative of solar-assisted CaL cycles.

Despite the technical advantages of using Ca-based sorbents to produce H_2_-rich syngas, the rapid loss of sorption capacity and the resulting need for continuous make-up with fresh sorbent remain the main bottlenecks of this technology. It is well known that CaO regeneration conditions play a critical role in the overall performance of the calcium looping (CaL) process. Depending on the specific operating parameters, deactivation of the sorbent due to pore blockage or sintering can progressively reduce CaO reactivity [10,11,12]. This results in a significant decline in the extent of carbonation over successive calcination/carbonation cycles, ultimately requiring the replacement of the spent sorbent. Various strategies have been explored to enhance sorbent durability, including improving structural stability addition of inert compounds with high Tamman temperatures to the sorbent structure (such as ZrO_2_/CaCO_3_ or Al_2_O_3_/CaCO_3_) [13,14,15], reactivating the sorbent via steam hydration treatments [5,16], enhancing the textural properties of CaO through advanced sorbent engineering approaches, and adding alkali salts to the sorbents [17,18,19].

However, the literature data have shown that the high temperature required for its decomposition is the main factor contributing to sorbent deactivation [20]. When CO_2_ sequestration is the goal, the calciner is typically operated under a pure CO_2_ atmosphere. Under atmospheric pressure, this can result in calcination temperatures as high as 950 °C, which accelerates the deactivation of the sorbent [11,21]. Therefore, reducing the calcination temperature has been identified as the most straightforward strategy to mitigate sorbent deactivation. This can be achieved by lowering the CO_2_ molar fraction in the calciner, typically through the addition of an inert gas such as N_2_. In the absence of CO_2_, the equilibrium temperature decreases, enabling calcination at temperatures as low as 750 °C. However, to enable CO_2_ sequestration, a gas separation unit is required, posing both economic and technical challenges due to the difficulty of separating CO_2_ from N_2_. Recently, a novel approach involving calcination under low absolute CO_2_ partial pressures (0.01–0.1 bar) has proven that calcination is feasible at temperatures as low as 650 °C [22,23].

The use of steam as a dilution gas during calcination offers several advantages [24,25,26,27,28]. As with any other dilution gas, the reduction in the partial pressure of CO_2_ enables calciner operation at lower temperatures. However, the presence of steam accelerates the calcination reaction, allowing the calciner to operate only slightly above the equilibrium temperature [26]. CaO regenerated in the presence of steam exhibits a less sintered microstructure and a more reactive surface, which significantly enhances the subsequent carbonation step and improves the multicycle CO_2_ sorption capacity [27]. Moreover, steam can be effectively separated from CO_2_ by condensation. Nonetheless, this method introduces a considerable energy and exergy penalty, as lowering the temperature to 800 °C may require more than half of the flue gas to consist of steam.

Although several works have explored the impact of CO_2_ partial pressure and inert gas dilution on CaO reactivity, most studies have been performed under dry atmospheres and at high temperatures (typically above 900 °C). Consequently, the combined effect of low CO_2_ partial pressures and high steam contents remains poorly understood. There is a lack of systematic data describing the kinetic behavior of natural Ca-based sorbents under mild temperatures and steam-rich conditions [7]. Addressing this gap is crucial for the design of efficient and durable CaL-based systems operating with renewable heat inputs.

Steam-assisted calcination of Ca-based sorbents has previously been investigated using thermogravimetric and micro-reactor techniques, where the influence of steam on CaCO_3_ decomposition kinetics was examined using small sample masses and in the absence of fluidized bed hydrodynamics [26,27,29,30]. These studies provide valuable mechanistic insight into the role of steam but were generally conducted at high temperatures and were not intended to explore calcination under mild, near-equilibrium conditions.

Fluidized bed studies incorporating steam during calcination have also been reported [25,28,31]. However, these investigations were performed under conventional calcium looping or thermochemical energy-storage calciner operating conditions, at temperatures ≥ 900 °C and elevated CO_2_ partial pressures, where calcination proceeds far from equilibrium and steam primarily acts to mitigate sintering during regeneration.

In contrast, the present work deliberately investigates the calcination kinetics of natural limestone and dolomite in a bubbling fluidized bed reactor under a combined operating window of mild temperatures (775–850 °C), low CO_2_ partial pressures (0.05–0.10 bar), and steam-rich atmospheres (90–95%vol H_2_O). In the case of dolomite-derived sorbents, the presence of MgO may also influence CO_2_–solid interactions during calcination–carbonation cycles, as MgO has been reported to participate in carbonate-related surface processes and to modify the local reaction environment, thereby affecting CO_2_ adsorption and reaction pathways [32,33]. This calcination regime is explored as a strategy to enable CO_2_ capture while minimizing sorbent deactivation under thermally moderate conditions, and is compatible with sorption-enhanced gasification and solar-assisted calcium looping configurations.

Furthermore, carbonation was carried out under sorption-enhanced gasification-representative conditions (650 °C and low CO_2_ partial pressure in H_2_–H_2_O-containing gas mixtures), allowing a consistent assessment of how calcination under steam-rich, mild conditions influences sorbent activity and deactivation over multiple carbonation–calcination cycles.

## 2. Material and Methods

### 2.1. Sorbent Characterization

Natural limestone of high purity (99.5 wt% CaCO_3_) and natural dolomite (94.45 wt% CaMg(CO_3_)_2_ and 5 wt% CaCO_3_) from Minera del Santo Angel quarry (Sevilla, Spain) were tested. Table 1 presents the physical and textural characterization of the limestone and dolomite samples used in this study. The average equivalent particle diameter was estimated from the particle size distribution measured by laser diffraction using a Mastersizer 2000 instrument (Malvern Panalytical Ltd., Malvern, UK). The true density was determined by helium pycnometry with a Quantachrome Pentapyc 5200e pycnometer (Anton Paar QuantaTec Inc., Boynton Beach, FL, USA). The BET surface area was measured by N_2_ adsorption using a Micromeritics ASAP 2420 analyzer (Micromeritics Instrument Corporation, Norcross, GA, USA).

Both materials, limestone and dolomite, were selected with a controlled particle size range between 250 and 355 µm, ensuring comparable conditions in terms of heat and mass transfer during the experiments. This specific range was chosen to prevent particle elutriation from the fluidized bed reactor and to better mimic the typical particle sizes used in industrial-scale feedstock for dual fluidized bed systems. Limestone exhibited a significantly higher BET surface area (0.44 m^2^/g) compared to dolomite (0.05 m^2^/g), suggesting a greater availability of reactive surface in the fresh state. However, dolomite showed a larger average pore diameter (39.03 nm vs. 20.98 nm), which may favor internal gas diffusion during the reaction. The specific surface area per unit volume (S_0_) was also higher for limestone, with a value of 19.05 × 10^7^ m^2^/m^3^, compared to 10.38 × 10^7^ m^2^/m^3^ for dolomite. To characterize the dolomite at the beginning of the calcination–carbonation cycles, a half-calcined dolomite sample was obtained. This sample was heated inside the reactor, using the same preheating method as during the calcination kinetics experiments, to ensure the decomposition of only MgCO_3_, but not CaCO_3_. As a result of the partial calcination treatment, the half-calcined dolomite exhibited noticeable changes in its physical and textural properties. The particle density decreased from 2878.4 to 2315.8 kg/m^3^ due to the partial mass loss associated with MgCO_3_ decomposition. In parallel, an increase in BET surface area (from 0.05 to 0.12 m^2^/g) and average pore diameter (from 39.03 to 58.50 nm) was observed, indicating the generation of additional intraparticle porosity during the selective decomposition of the MgCO_3_ phase. Despite the increase in porosity, the specific surface area per unit volume (S_0_) decreased, mainly because of the lower particle density. These properties are representative of dolomite at the onset of calcination–carbonation cycles, where MgO is already present while CaCO_3_ remains largely undecomposed.

### 2.2. Experimental Setup

**Figure 1:**
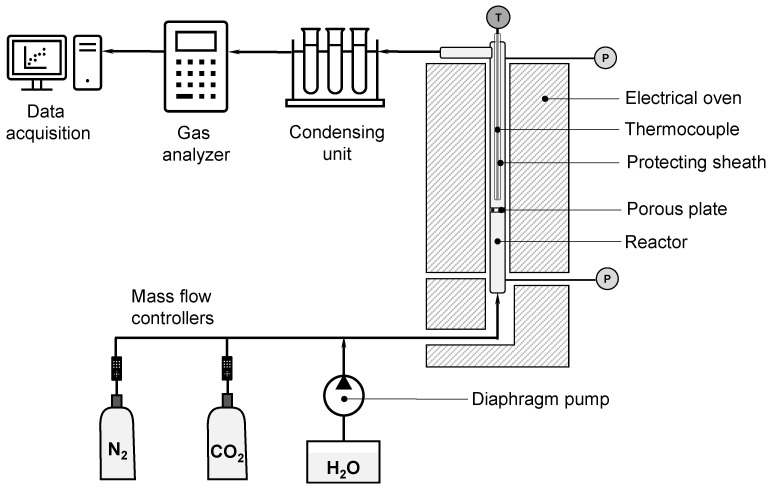
Experimental setup.

The laboratory fluidized bed (FB) experimental setup consists of a cylindrical quartz reactor operated at atmospheric pressure. The FB has an inner diameter of 32 mm and is surrounded by an electrical oven. The reactor is equipped with a porous plate that supports the sorbents and serves as a gas distributor. A thermocouple is situated inside the reactor at approximately 3 cm above the static bed height of the solids and is employed to control the reaction temperature. The part of the thermocouple that is located inside the reactor is protected by a protecting sheath made of quartz, to avoid direct contact between the gas flow and the thermocouple. A schematic representation of the experimental setup is shown in Figure 1.

During the experimental phase for calcination–carbonation cycles, various types of gases were used: N_2_, CO_2_ and H_2_O. The flowrates of N_2_ and CO_2_ fed to the reactor were measured by mass flow controllers. The H_2_O flow was controlled by a diaphragm liquid dosing pump operating with deionized water. The steam was generated and mixed with the other gases in a preheating zone located below the gas distributor. The CO_2_ concentration in the gas was measured with a non-dispersed infrared analyzer.

### 2.3. Experimental Conditions

**Figure 2:**
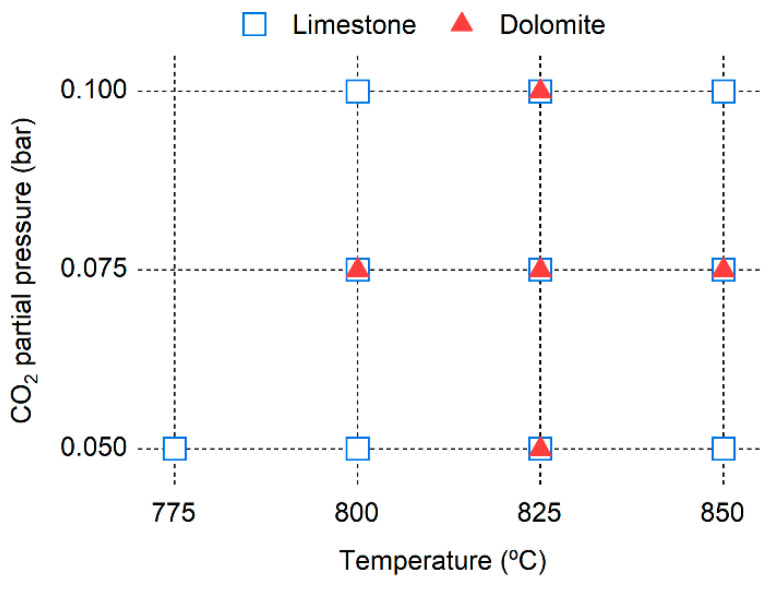
Operating conditions employed in the calcination tests.

The operating conditions tested during cyclic calcination–carbonation cycles are summarized in Figure 2. The total pressure during the experiments was close to 1 atm. Calcination tests were conducted at temperatures between 775 and 850 °C and using CO_2_ partial pressures of 0.05, 0.075, and 0.10 bar, with H_2_O being the balance gas. The experiments were carried out over 10 calcination–carbonation cycles. Carbonation was conducted at 650 °C and with a CO_2_ partial pressure of 0.05 bar and an H_2_O partial pressure of 0.50 bar, with N_2_ used as the balance gas. These carbonation conditions were selected to resemble the composition of a syngas produced in a steam-blown fluidized bed gasifier (with in situ CO_2_ capture) in a SEG dual FB system [34]. Additional experiments were conducted using limestone at 800 °C to assess the influence of steam concentration during the first calcination cycle. During these carbonation tests, the CO_2_ partial pressure was maintained constant at 0.05 bar and the steam pressure varied between 0 and 0.95 bar, with N_2_ being the balance gas. The total pressure in the bed was close to atmospheric pressure during the experiments. The calcium balance of the reactions was verified in all the tests. The superficial gas velocity at the reactor inlet was between 2 and 3 times the minimum fluidization velocity, ensuring stable bubbling fluidization with effective gas–solid contact. Under these conditions, external mass transfer resistances are minimized. In addition, the relatively small particle size (250–355 μm) and the high heat transfer coefficients, characteristic of fluidized beds, support the assumption of negligible external mass and heat transfer limitations under the operating conditions employed [35]. The initial batch of sorbent added to the reactor was small enough to ensure quasi-differential conversion in the reactor.

### 2.4. Experimental Procedure

In the experiments performed, the FB reactor was initially loaded with 6 g of the sorbent sample. The experimental procedure consisted of the following steps:

Heating: The reactor temperature increased up to the set test temperature at a rate of 20 °C/min. During the heating, pure CO_2_ was fed into the FB to prevent decomposition of CaCO_3_.

Calcination: Once the calcination temperature was reached, the gas was switched to the calcination atmosphere selected for each experiment. The temperature was maintained until the sorbent calcination was complete, i.e., when the outlet CO_2_ concentration returned to its initial inlet value and stayed at that value for 5 min.

Cooling: when the sorbent calcination was completed, the feed gas was switched to pure N_2_ and the reactor was cooled until reaching the carbonation temperature, set at 650 °C.

Carbonation: at a constant temperature of 650 °C, the gas was switched to the carbonation mixture previously described and these conditions were maintained until the CO_2_ concentration rose to its initial value and for an additional 5 min after that.

Finally, the previously described steps were repeated to complete a total of 10 calcination–carbonation cycles. At the end of the carbonation step in the tenth cycle, the sample was cooled under a pure CO_2_ atmosphere to avoid CaCO_3_ calcination and allow for verification of the calcium mass balance.

The measured gas concentrations were corrected to account for the dispersion in the gas outlet line. Blank tests with CO_2_ injection into the fluidized bed were performed and it was found that the system could be well characterized by a first-order response with a time constant equal to 4.8 s [36]. During the calcination–carbonation tests carried out in this study, reaction (1) takes place. Therefore, the total degree of conversion (α) of the sorbent was calculated based on the CO_2_ outlet concentration. However, it should be noted that during the heating stage of dolomite, MgO is also formed [27]. This phase must be considered in the mass balance, even though it does not participate as a reactive component during the calcination–carbonation cycles.

(1) $$CaC{O_{3}}_{\left(s\right)}⇄{CaO}_{\left(s\right)}+{{CO}_{2}}_{\left(g\right)}$$

## 3. Results and Discussion

### 3.1. Effect of CO_2_ Partial Pressure and Temperature on the Calcination Rate During the Initial (First) Cycle

**Figure 3:**
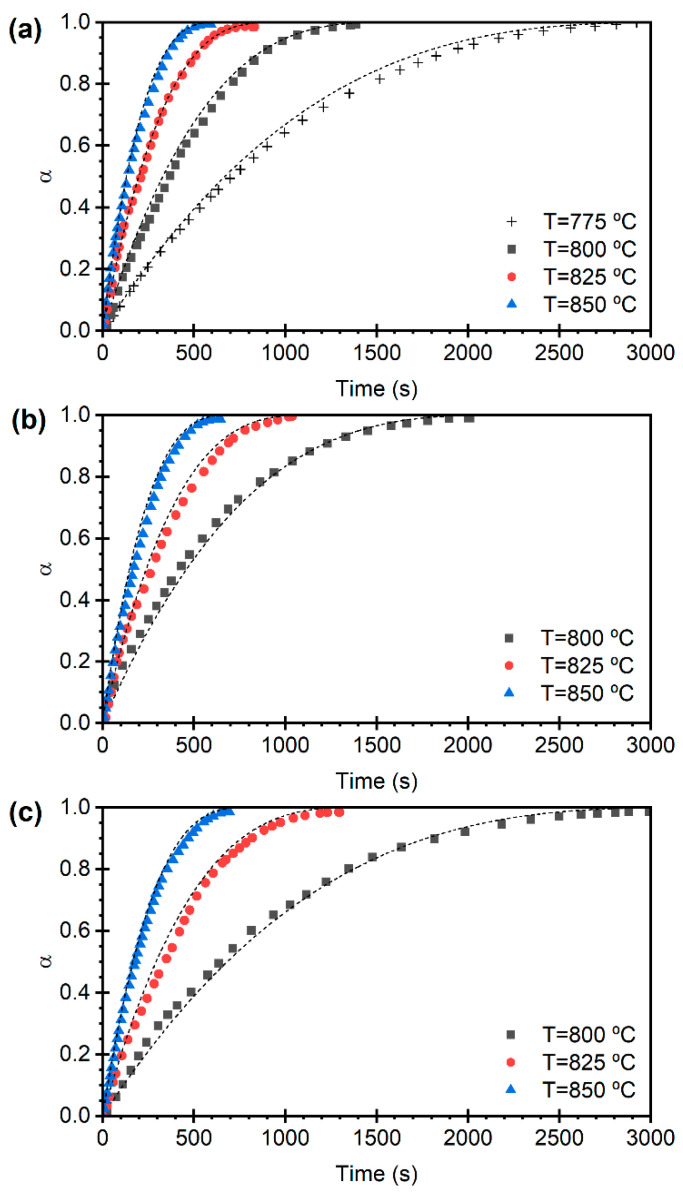
Temporal evolution of the degree of conversion during limestone calcination in the first cycle, measured at different temperatures and using different CO_2_ partial pressures: $p_{CO_{2}}$ = 0.05 bar (**a**); $p_{CO_{2}}$ = 0.075 bar (**b**); and $p_{CO_{2}}$ = 0.10 bar (**c**). Model predictions in dashed lines according to Equations (8) and (9).

Figure 3 shows the temporal evolution of the limestone conversion degree at different temperatures, varying between 775 and 850 °C, and CO_2_ partial pressures of the CO_2_-H_2_O mixtures, ranging from 0.05 to 0.10 bar. As expected, higher reaction rates are observed with increasing temperature for each CO_2_ partial pressure. This behavior is attributed both to kinetics and to the rise in the equilibrium CO_2_ partial pressure with temperature, which increases the driving force for the calcination reaction, as described in Equation (4). For instance, at $p_{CO_{2}}$ = 0.05 bar (see Figure 3a), the calcination reaction is completed in approximately 2900 s at 775 °C, whereas at 850 °C, the reaction time is reduced to just 570 s. This trend is also observed for the other CO_2_ partial pressures (Figure 3b,c).

In contrast, at constant temperature, an increase in CO_2_ partial pressure leads to a slower calcination reaction. This is due to the reduction in the driving force for the reaction, since higher CO_2_ partial pressures approach the equilibrium value, thereby limiting the decomposition of CaCO_3_. For example, at 800 °C, the time required to complete calcination increases from 1380 s at $p_{CO_{2}}$ = 0.05 bar to 2990 s at $p_{CO_{2}}$ = 0.10 bar. A similar trend is observed at 850 °C, where the calcination time increases by 17% as the CO_2_ partial pressure increases from 0.05 to 0.10 bar. These results confirm the strong influence of CO_2_ partial pressure on the decomposition kinetics of limestone under isothermal conditions.

**Figure 4:**
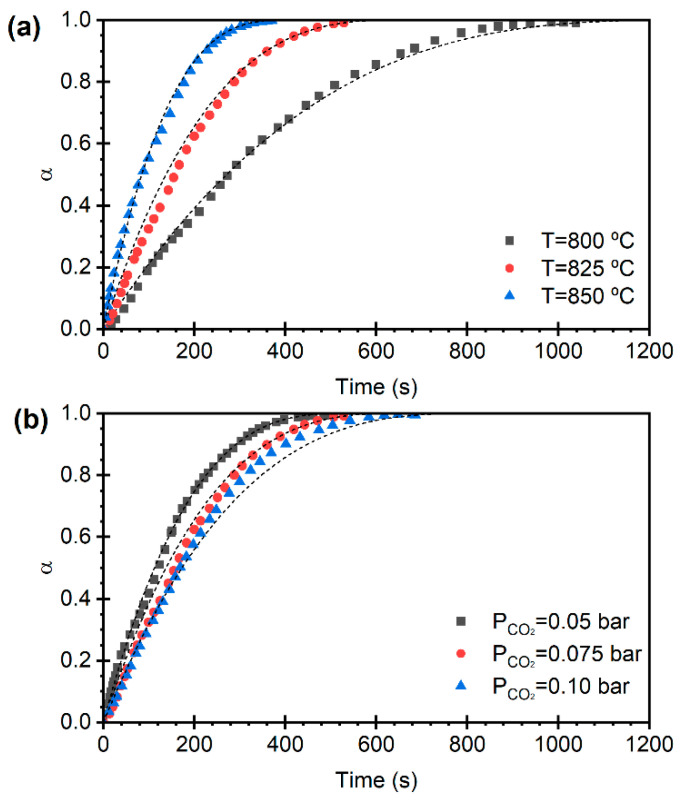
Temporal evolution of the degree of conversion during dolomite calcination in the first cycle with $p_{CO_{2}}$ = 0.075 bar and for different temperatures (**a**), and for a calcination temperature of 825 °C, using different CO_2_ partial pressures (**b**). Model predictions in dashed lines according to Equations (8) and (10).

As shown in Figure 4, dolomite exhibits qualitatively similar behavior to limestone during calcination: higher temperatures result in faster reaction (Figure 4a), while increasing the CO_2_ partial pressure at constant temperature (Figure 4b) has the opposite effect. However, dolomite showed higher calcination rates compared to limestone. For example, at a temperature of 825 °C and a CO_2_ partial pressure of 0.075 bar, limestone reached complete conversion after 1040 s, whereas dolomite required only 528 s. The faster calcination of dolomite compared to limestone is in agreement with results from the literature [34,37,38]. This can be explained by the highly porous solid matrix generated from the decomposition of MgCO_3_ during the heat up of the sample [38], generating a larger surface area available for reaction, thus enhancing the reaction rate [25].

The experimental results obtained in this study exhibit a behavior consistent with previous works regarding the influence of CO_2_ partial pressure and temperature on calcination. However, the present work reveals a slower reaction rate than expected when compared to values reported by other authors [26,31,39]. This discrepancy is attributed to the difference in the properties of the sorbents evaluated, especially the particle size, where the literature in the conditions of interest has worked with particles between 20 and 120 µm.

### 3.2. Influence of Calcination Conditions and Cyclability on the Carbonation Process

**Figure 5:**
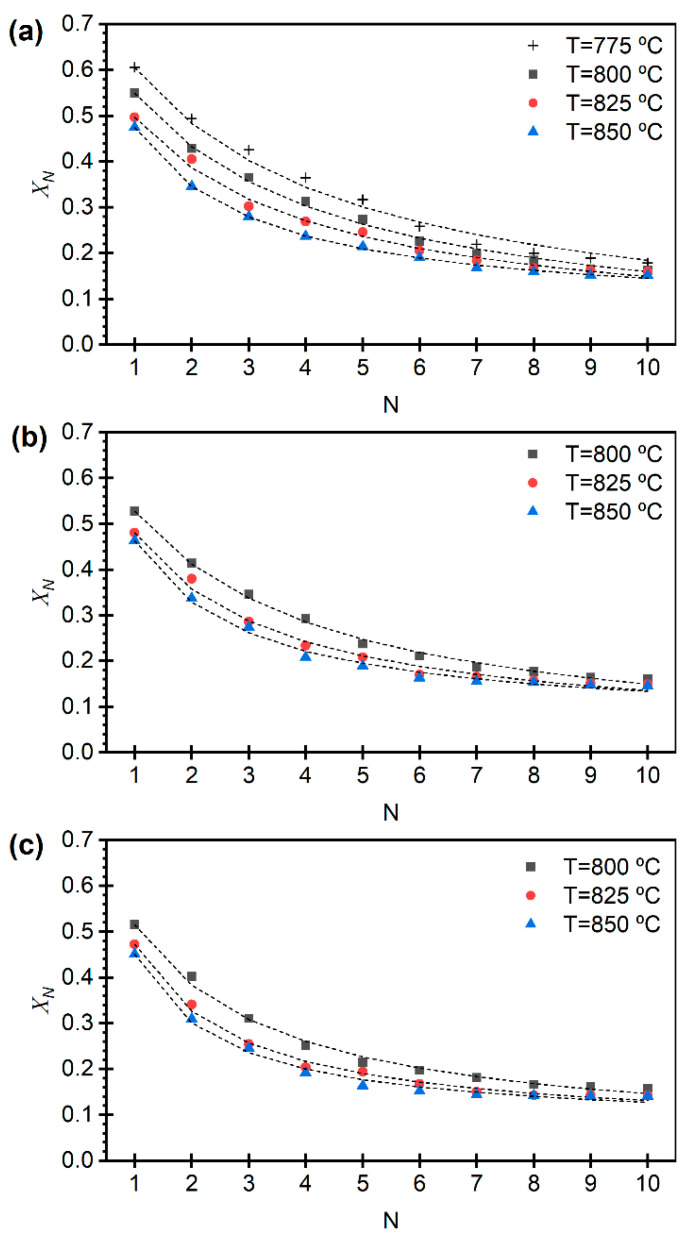
Cycle evolution of *X_N_*, the effective carbonation conversion for the different limestone calcination conditions, with $p_{CO_{2}}$ = 0.05 bar (**a**), $p_{CO_{2}}$ = 0.075 bar (**b**), and $p_{CO_{2}}$ = 0.10 bar (**c**). Carbonation conditions are T = 650 °C, $p_{CO_{2}}$ = 0.05 bar, $p_{N_{2}}$ = 0.45 bar and $p_{H_{2}O}$ = 0.50 bar. Model predictions in dashed lines according to Equation (2).

**Figure 6:**
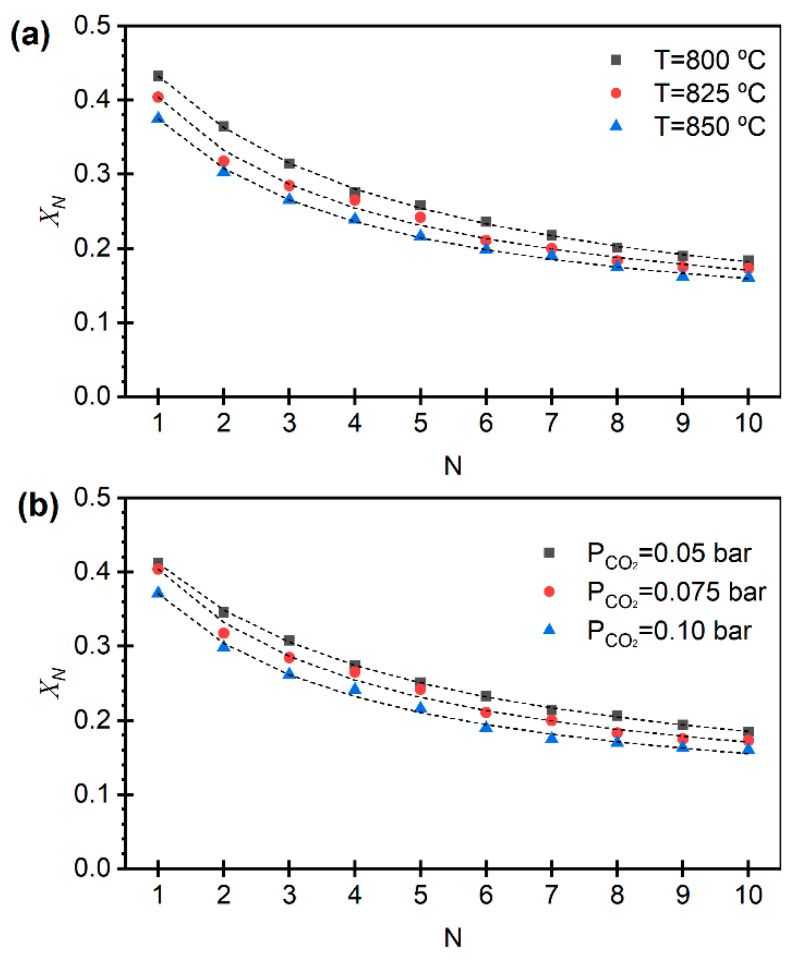
Cycle evolution of $X_{N}$, the effective carbonation conversion for the different dolomite calcination conditions, with $p_{CO_{2}}$ = 0.075 bar and varying temperature (**a**), and calcination temperature of 825 °C and varying $p_{CO_{2}}$ (**b**). Carbonation conditions are T = 650 °C, $p_{CO_{2}}$ = 0.05 bar, $p_{N_{2}}$ = 0.45 bar and $p_{H_{2}O}$ = 0.50 bar. Model predictions in dashed lines according to Equation (2).

Figure 5 and Figure 6 show the evolution of the effective carbonation conversion, i.e., the final degree of conversion of CaO to CaCO_3_, reached in the carbonation stage, referred to the total Ca in the sample, over 10 calcination–carbonation cycles (N) under different calcination conditions for limestone and dolomite, respectively. The carbonation conditions were kept constant throughout all experiments. For the two sorbents and for all calcination conditions tested, a progressive decline in the CO_2_ sorption capacity was observed, attributed to the loss of CaO reactivity due to sintering. This deactivation becomes more pronounced with increasing temperature and CO_2_ partial pressure during calcination, as these conditions intensify sintering processes and reduce the accessibility of reactive sites [8,12].

Conversely, under milder calcination conditions, more stable conversion performance can be maintained. This effect is more evident in the case of dolomite, which benefits from the presence of MgO acting as a structural stabilizer that inhibits the densification of reactive CaO [9,40,41]. As shown in Figure 5, limestone exhibits higher effective carbonation conversion during the initial cycles; however, the absence of a stabilizing component such as MgO leads to rapid deactivation due to sintering, until a stable carbonation conversion is reached. In contrast, although dolomite shows lower initial CO_2_ sorption capacity due to its high content of non-reactive MgO, it displays greater stability over successive cycles, reflecting a structure more resistant to degradation [41].

As previously discussed, the materials under study are expected to undergo a large number of cycles under real process conditions before being replaced. In this context, Equation (2) provides a semi-empirical expression that effectively fits the multicycle conversion data [27].

(2) $$X_{N}=\frac{X_{1}}{\kappa \left(N-1\right)+{\left(1-\frac{X_{r}}{X_{1}}\right)}^{-1}}+X_{r}$$

where *X_N_* is the effective carbonation conversion after cycle N, $X_{1}$ the effective conversion at the first cycle, N the evaluated cycle, *κ* the deactivation constant and $X_{r}$ the residual conversion, the constant $X_{N}$ value reached after a very large number of cycles.

The dashed lines in Figure 5 and Figure 6 were obtained using the expression given by Equation (2), with the parameters fitted by least-squares minimization, as presented in Table 2. An excellent agreement is observed between experimental data and the modeled values. The parameter that provides the most insight into the phenomena studied is the deactivation constant *κ*, which increases under more severe calcination conditions prior to the carbonation stage (i.e., longer times, higher CO_2_ partial pressures, and elevated temperatures).

The lower deactivation constants obtained for dolomite relative to limestone can be quantitatively interpreted as a slower loss of reactive CaO surface area with cycling, consistent with the stabilizing role of MgO in dolomite-derived sorbents. Although no direct structural characterization of the sorbents after repeated calcination–carbonation cycles was conducted in this work, the observed differences in calcination kinetics and cyclic behavior between limestone and dolomite can be discussed on the basis of the experimental results and well-established structural mechanisms reported in the literature. The MgO phase formed during MgCO_3_ decomposition acts as a thermally stable, inert component with a significantly higher Tamman temperature than CaO, inhibiting CaO–grain coalescence during calcination and repeated cycling. X-ray diffraction and electron microscopy studies have shown that the presence of MgO suppresses CaO crystallite growth and reduces sintering rates compared to limestone-derived CaO, leading to improved retention of reactive surface area over multiple cycles [40,41,42]. This effect provides a direct mechanistic explanation for the lower values of the deactivation constant κ observed for dolomite in the present work.

Moreover, the early decomposition of MgCO_3_ generates a porous MgO-rich solid matrix prior to CaCO_3_ calcination, which provides structural support to the CaO phase formed in subsequent steps. This effect is already evident at the half-calcined stage, as reflected by the textural properties reported in Table 1 for half-calcined dolomite, which indicate a more open solid structure compared to limestone. Such a MgO-containing matrix stabilizes pore architecture and increases resistance to pore collapse during the cyclic calcination–carbonation operation. Previous studies based on BET surface area measurements and mercury porosimetry have shown that dolomite maintains larger average pore diameters and improved pore connectivity than limestone under calcium looping conditions [9,12,41]. From a kinetic standpoint, this enhanced structural robustness limits the progressive loss of accessible CaO reaction sites and contributes to the reduced deactivation rates quantified by κ.

Finally, MgO has been shown to reduce surface diffusion of Ca^2+^ species and to delay densification of the CaO phase during thermal cycling. Investigations of MgO–CaO systems indicate that MgO interferes with oriented CaO crystal growth, resulting in a more heterogeneous and sinter-resistant microstructure [40,42]. This cumulative effect becomes increasingly relevant with the number of cycles and is therefore consistently reflected in the lower deactivation constants measured for dolomite across the range of calcination conditions explored in this study.

In addition to the deactivation constant κ, the residual conversion ($X_{r}$) provides important insight into the long-term performance of the sorbents. While κ characterizes the rate of deactivation, $X_{r}$ represents the asymptotic CO_2_ uptake capacity that remains after a large number of cycles and is therefore directly related to the ultimate sorbent utilization.

As shown in Table 2, limestone exhibits very low $X_{r}$ values under most conditions, approaching zero at lower temperatures and CO_2_ partial pressures, which indicates a near-complete loss of CO_2_ uptake capacity after prolonged cycling. In contrast, dolomite consistently shows higher $X_{r}$ values (≈0.06–0.08), indicating the preservation of a residual active fraction even after multiple cycles.

This behavior highlights the enhanced structural stability of dolomite-derived sorbents and is consistent with the presence of the MgO matrix, which limits sintering and helps maintain a fraction of accessible CaO. From a process perspective, these differences in $X_{r}$ are highly relevant, as they directly impact the long-term sorbent replacement rate and overall CO_2_ capture efficiency.

### 3.3. Kinetic Modeling

Previous works have suggested that for particle sizes and sorbent porosities similar to the materials employed here, the calcination reaction proceeds by kinetic control in a narrow reaction front separating the unreacted CaCO_3_ core from the CaO forming in an external shell, whose thickness increases with time [34,38]. The other extreme model describing a uniform conversion throughout the particle is typically applied for particle sizes below 100 µm. Figure 3 and Figure 4 show that both limestone and dolomite exhibit an initial fast kinetic regime, followed by a transition to a slightly slower reaction rate, which can be described by the shrinking core model, with kinetic control, at least during the initial stage of calcination. Although the recent literature has proposed more complex particle conversion models, such as the Random Pore Model (RPM), which can account for slow and transitional reaction regimes, in this work, the experimental data were fitted using the SCM due to its simplicity, mathematical tractability, and the good agreement observed with the calcination curves, even when considering their evolution over multiple cycles.

Given the particle size range used (250–355 μm), it is reasonable to assume spherical geometry, and the particle size is considered to be constant throughout the reaction. According to the kinetically controlled shrinking core model, the solid conversion rate can be expressed by Equation (3).

(3) $${\left(\frac{d\alpha }{dt}\right)}_{N=1}=\frac{1}{d_{p}}A_{0}\exp\left(-\frac{E_{a}}{RT}\right)f\left(p_{CO_{2}}\right)\;3{\left(1-\alpha \right)}^{2/3}\;$$

$A_{0}$ is the pre-exponential factor; $d_{p}$ is the particle diameter (and $E_{a}$ is the activation energy). $f\left(p_{CO_{2}}\right)$ is a function that expresses the dependence of the calcination kinetics on the CO_2_ partial pressure, which has been modeled using the expression given by Equation (4).

(4) $$f\left(p_{CO_{2}}\right)={\left(1-\frac{p_{CO_{2}}}{p_{CO_{2},eq}}\right)}^{n}$$

This equation is based on the difference between the equilibrium pressure $p_{CO_{2},eq}\;$ and the operating partial pressure $p_{CO_{2}}$. The exponent, n, is the corresponding reaction order, with respect to CO_2_. This equation has been employed to model the calcination kinetics in previous works [43].

The equilibrium partial pressure of CO_2_, $p_{CO_{2},eq}$, depends on the calcination temperature and can be determined using Equation (5) [44].

(5) $$p_{CO_{2},eq}=4.137\times 10^{7}\exp\left(-\frac{20474}{T}\right)$$

The values of $A_{0}$, $E_{a}$ and n that describe the calcination of limestone and dolomite have been obtained by fitting the initial slopes of the corresponding α vs. t curves ${\left(\frac{d\alpha }{dt}\right)}_{N=1}$ using a nonlinear regression model, and Equation (6) was obtained expressing the calcination rate of limestone.

(6) $${\left(\frac{d\alpha }{dt}\right)}_{N=1}=\frac{1}{d_{p}}2.21\times 10^{5}\exp\left(-\frac{120.27}{RT}\right)\;{\left(1-\frac{p_{CO_{2}}}{p_{CO_{2},eq}}\right)}^{2.01}\;3{\left(1-\alpha \right)}^{2/3}$$

Under the same assumptions, the dolomite calcination conversion as a function of time can be expressed by Equation (7).

(7) $${\left(\frac{d\alpha }{dt}\right)}_{N=1}=\frac{1}{d_{p}}2.60\times 10^{5}\exp\left(-\frac{115.96}{RT}\right)\;{\left(1-\frac{p_{CO_{2}}}{p_{CO_{2},eq}}\right)}^{2.19}\;3{\left(1-\alpha \right)}^{2/3}$$

The selection of the shrinking core model is supported by previous experimental studies on natural limestone and dolomite, which have shown that calcination proceeds with a well-defined reaction front and a core–shell morphology for particle sizes comparable to those employed here, and that the progressive decrease in reaction rate with conversion can be attributed to geometric shrinkage of the unreacted core rather than to a change in reaction mechanism. Moreover, for particles in this size range, the shrinking core model has been shown to provide good agreement with experimental calcination conversion–time data under a wide range of operating conditions [34,38,39,45].

The obtained equations accurately represent the experimental data, with a coefficient of determination R^2^ of 97.98% for limestone calcination and 95.31% for dolomite calcination. The integrated forms of the expressions are presented in Equation (8).

(8) $$\alpha =1-{\left(1-\frac{t}{\tau_{S}}\right)}^{3}$$

The time required to reach complete calcination (*τ_S_*) for limestone and dolomite can be determined using Equations (9) and (10), respectively.

(9) $$\tau_{S}=\frac{1}{\frac{1}{d_{p}}2.21\times 10^{5}\exp\left(-\frac{120.27}{RT}\right)\;{\left(1-\frac{p_{CO_{2}}}{p_{CO_{2},eq}}\right)}^{2.01}\;\;}$$

(10) $$\tau_{S}=\frac{1}{\frac{1}{d_{p}}2.60\times 10^{5}\exp\left(-\frac{115.96}{RT}\right)\;{\left(1-\frac{p_{CO_{2}}}{p_{CO_{2},eq}}\right)}^{2.19}\;\;}$$

The dashed lines in Figure 3 and Figure 4 represent the results obtained from Equations (8)–(10), using the determined kinetic parameters. These curves show a reasonably good fit between the calculated conversion degree as a function of time and the experimental data.

The experimentally determined activation energy for limestone was 120.27 kJ/mol, which falls within the range of kinetic parameters reported in the literature for CaCO_3_ decomposition. This process has been extensively studied under various operating conditions, primarily using thermogravimetric analysis, with reported activation energies ranging from 86 to 322 kJ/mol [39,46,47,48,49]. Kinetic studies of calcination in fluidized bed reactors are less common, but reported values typically fall within the “lower range”, between 100 and 170 kJ/mol [34,50]. Activation energies in a steam atmosphere have been reported to be significantly lower than in dry conditions, which has been attributed to a different reaction pathway under these conditions [26,27].

For dolomite calcination, activation energy of 115.96 kJ/mol was obtained, which lies within the range of 113–149 kJ/mol reported in previous experimental studies [30,38,51,52]. The lower apparent activation energy observed for dolomite is attributed to the MgO solid-state matrix, which alters the reaction mechanism by both destabilizing the CaCO_3_ nanocrystals and providing heterogeneous nucleation sites for CaO formation [42]. As with limestone, the presence of steam has been reported to enhance the decomposition kinetics of dolomite, as well as its sintering resistance over multiple cycles [29,31,44].

The comparison of activation energies was assessed based on their expected 95% confidence intervals. Considering the typical uncertainties associated with Arrhenius-based estimations, the difference between the values obtained for limestone (120.27 kJ/mol) and dolomite (115.96 kJ/mol) is relatively small and likely falls within experimental error. Therefore, no statistically significant difference can be established. Nevertheless, the slightly lower activation energy observed for dolomite is consistent with the mechanistic role of the MgO matrix reported in the literature.

**Figure 7:**
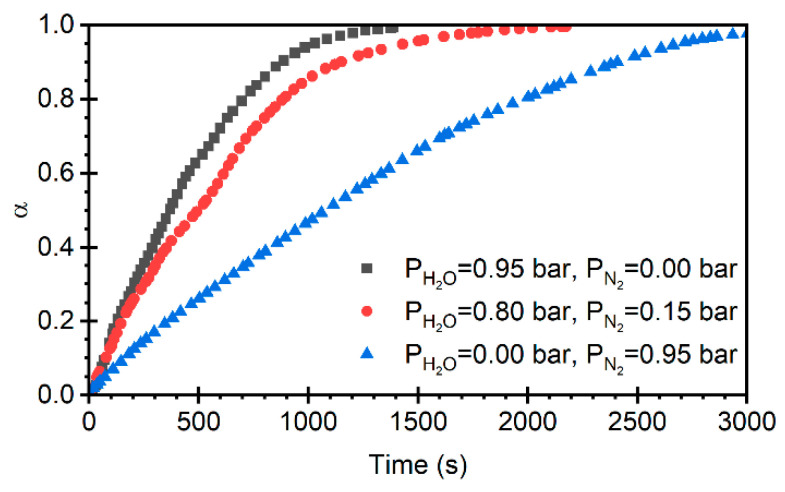
Effect of steam partial pressure on the degree of conversion during limestone calcination with $p_{CO_{2}}$ = 0.05 bar and T = 800 °C.

To evaluate the effect of H_2_O partial pressure on the calcination reaction, experiments using only limestone were conducted at a CO_2_ partial pressure of 0.05 bar and a temperature of 800 °C, varying the $p_{H_{2}O}$ and using N_2_ as the balance gas (see Figure 7). It was observed that increasing the water vapor partial pressure during calcination led to up to a 50% reduction in the time required for complete particle conversion, compared to the test using only N_2_ and CO_2_. This confirms the pseudo-catalytic or acceleration effect of steam in calcium looping systems [27].

The acceleration of calcination kinetics in the presence of steam can be interpreted in terms of several surface-mediated mechanisms that have been proposed and discussed in the literature.

Steam promotes partial hydroxylation of CaCO_3_ and newly formed CaO surfaces, leading to the formation of transient surface hydroxyl species. These surface modifications weaken CaCO_3_ bonds at the reaction interface and reduce the energetic barrier associated with CO_2_ desorption, thereby lowering the apparent activation energy for calcination. This effect has been consistently reported in kinetic studies of CaCO_3_ decomposition in steam-containing atmospheres and is supported by both experimental observations and theoretical analyses [26,27,29]. The operation of this mechanism is fully consistent with the significantly higher initial calcination rates observed in the present work under steam-rich conditions, as shown in Figure 7.

In addition, steam has been reported to influence CaO nucleation and early crystal growth during calcination. In the presence of H_2_O, CaO tends to nucleate at a higher density of sites and initially forms smaller or less ordered crystallites, which delays the development of a dense and passivating CaO product layer at the CaCO_3_/CaO interface. Such steam-induced modifications to CaO nucleation pathways have been proposed to enhance accessibility of the reaction front and are particularly relevant under near-equilibrium calcination conditions [37,45]. As with surface hydroxylation, this mechanism primarily affects the early stages of calcination and provides an additional explanation for the enhanced initial reaction rates measured in the present study under low CO_2_ partial pressure and steam-rich atmospheres.

Finally, although not directly quantified in the present experiments, steam has been shown in the literature to mitigate early-stage sintering of CaO during calcination and cyclic operation. By reducing surface diffusion and delaying densification of the product layer, the presence of steam helps preserve reactive surface area and pore accessibility over repeated calcination–carbonation cycles [28,31]. This mechanism predominantly influences the multicycle evolution of sorbent capacity, rather than the intrinsic initial calcination rate. Consistent with this interpretation, previous studies on steam-assisted calcium looping and thermochemical energy-storage systems have reported improved cyclic stability when steam is present during calcination [27].

Regarding the reaction order n, similar values were obtained for both sorbents: n = 2.01 for limestone and n = 2.19 for dolomite. These values indicate that the process is sensitive to small variations in CO_2_ partial pressure. When n > 1, a larger deviation from equilibrium is required to achieve appreciable reaction rates. For calcination processes of CaCO_3_ and CaMg(CO_3_)_2_ under conditions not limited by external diffusion, values of n ranging between 1 and 2.5, depending on the CO_2_ partial pressure and the conversion range used for kinetic fitting, have been reported [30,38,45]. Reaction orders with respect to CO_2_, larger than 1, have been attributed to the chemisorption of CO_2_ on the CaCO_3_ surface [38].

### 3.4. Effect of Cyclability on the Kinetics of the Calcination Reaction

**Figure 8:**
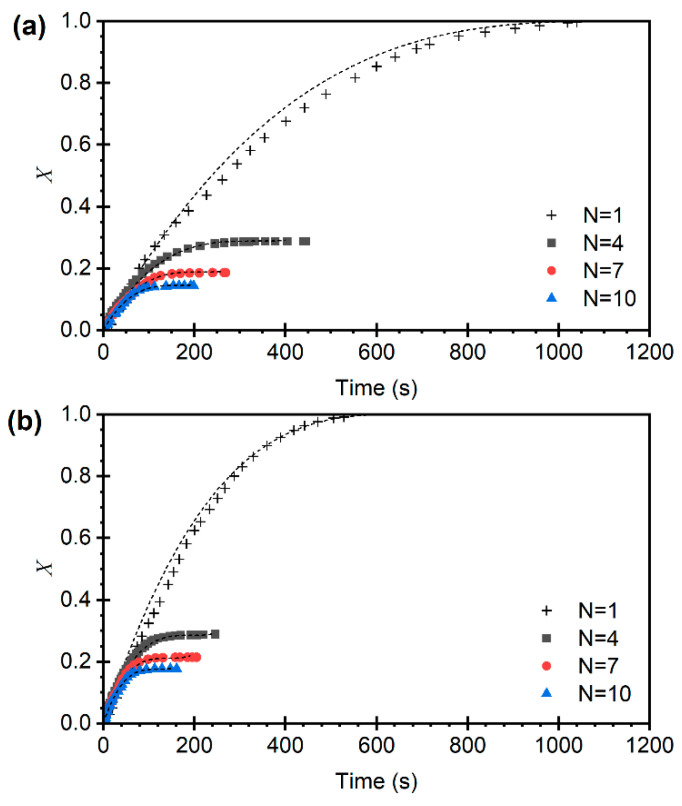
Conversion vs. time for different cycle numbers at 825 °C and $p_{CO_{2}}$ = 0.075 bar. Limestone (**a**) and dolomite (**b**). Model predictions in dashed lines according to Equation (11).

The calcination conversion curves as a function of time, obtained from the multicycle experiments, are presented in Figure 8. Considering that X represents the moles of CaCO_3_ calcined per mole of Ca, when the feedstock consists of pure CaCO_3_ entering the calcination process, X varies between 0 and 1. This condition is similar to that of the first cycle (*N* = 1), where α and X essentially coincide. In contrast, when the sorbent has been previously carbonated, the value of X varies between 0 and $X_{N-1}$ [31,39]. It can also be observed in Figure 8 that the reaction rates of the calcination process, determined as the initial slope of the X vs. t curves (dX/dt), remain constant across cycles at a given temperature and CO_2_ partial pressure.

While pore occlusion and sintering may occur simultaneously during cyclic operation, the nearly constant initial calcination rates observed over successive cycles indicate that the intrinsic reactivity of the CaCO_3_ formed in each carbonation step is preserved. This behavior is more consistent with a progressive, irreversible loss of active surface area, reducing the maximum extent of carbonation, than with dominant pore blockage effects, which would be expected to hinder gas access and affect the initial reaction rates as cycling proceeds.

The conversion models of the limestone and dolomite particles presented in Section 3.1 and Section 3.3 are normalized, that is, they are based on the amount of CaCO_3_ entering each calcination cycle. However, to provide evidence of deactivation with the number of cycles, the reaction rate must be expressed per mole of Ca according to Equations (6) and (8). This expression includes the variable $X_{N-1}$, which represents the upper limit of the moles of CaCO_3_ per mole of Ca that can be calcined in cycle N, as a result of the carbonation in the immediately preceding cycle; that is, for the calcination of cycle N, Equation (2) must be evaluated at N−1.

(11) $$\frac{dX}{dt}=A_{0}\exp\left(-\frac{E_{a}}{RT}\right){\left(1-\frac{p_{CO_{2}}}{p_{CO_{2},eq}}\right)}^{n}\;3{\left(1-\frac{X}{X_{N-1}}\right)}^{2/3}\;$$

**Figure 9:**
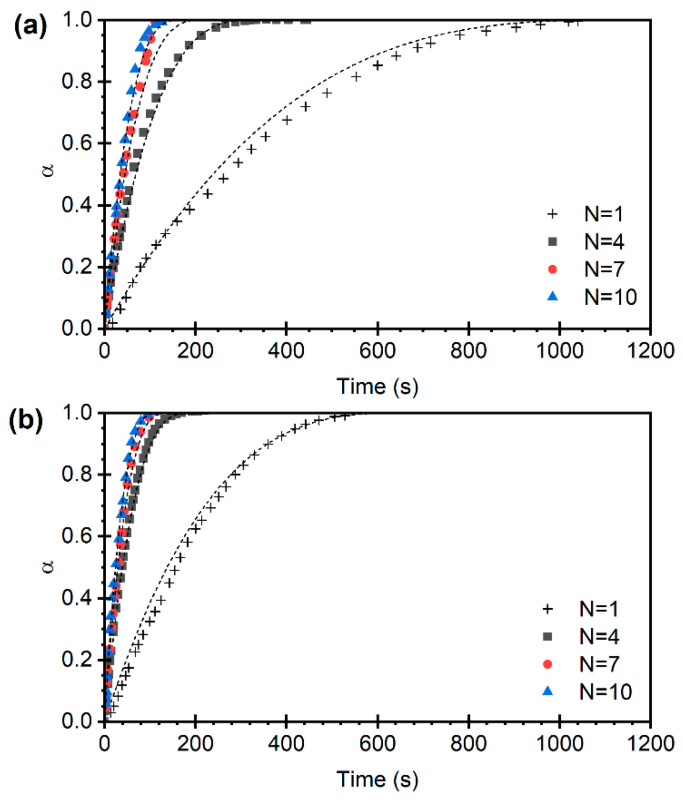
Normalized conversion vs. time for different numbers of calcination–carbonation cycles at 825 °C and $p_{CO_{2}}$ = 0.075 bar. Limestone (**a**) and dolomite (**b**). Model predictions in dashed lines according to the normalized Equation (11).

The dashed lines in Figure 8 show the results of the kinetic model obtained in Section 3.2, adapted using Equation (11), demonstrating good agreement between the model predictions and the experimental data. This behavior is also observed under all evaluated conditions of CO_2_ partial pressure and temperature. To better visualize the dimensional agreement between the model and the experiments, the normalized conversion is presented in Figure 9.

Figure 8 shows the loss of carbonation capacity of the sorbent with increasing number of calcination–carbonation cycles, which leads to a reduced effective mass of carbonate available for calcination. Consequently, less material actively participates in the reaction, and the system tends toward a stable conversion well below unity [20,41]. The practically constant initial reaction rate with increasing number of cycles observed in Figure 8 indicates that, although less CaCO_3_ is formed in each carbonation cycle, its reactivity is practically constant. This shows that deactivation is caused by an irreversible loss of active surface area inside the pores rather than pore occlusion.

The results obtained allow for the characterization of the calcination kinetics of both limestone and natural dolomite under experimental conditions representative of CO_2_ capture processes under multiple calcination–carbonation cycles.

## 4. Conclusions

The cyclic calcination–carbonation behavior of limestone and dolomite under conditions relevant for calcium looping gasification under fluidized bed (FB) conditions was evaluated. Experiments were conducted in a laboratory-scale FB reactor using CO_2_-H_2_O mixtures with CO_2_ partial pressures between 0.05 and 0.10 bar, at relatively low temperatures between 775 and 850 °C and at atmospheric pressure. The evolution of the total CaO carbonation capacity and the calcination kinetics over successive calcination–carbonation cycles was evaluated. A kinetic model was formulated and validated against experimental data. The calcination of limestone was observed to be much faster in an H_2_O atmosphere compared to using N_2_ as a fluidizing gas.

A progressive decline in effective conversion was observed over successive calcination–carbonation cycles, attributable to the loss of CaO reactivity due to sintering. This deactivation is more pronounced under harsher calcination conditions, with higher temperatures and CO_2_ partial pressures that promote material densification and reduce accessibility of reactive sites. In contrast, under milder conditions, conversion stability improves markedly, especially for dolomite, where the presence of MgO acts as a structural stabilizer.

Although the total sorption capacity was significantly reduced with increasing number of cycles, the rate of calcination of the active material was maintained approximately constant.

It was found that both materials can be accurately described using the shrinking core model (SCM) under kinetic control, with the derived kinetics providing good agreement across all evaluated cycles. The activation energies obtained were 120.27 kJ/mol for limestone and 115.96 kJ/mol for dolomite, consistent with previous studies conducted in the presence of steam. Additionally, the reaction order that describes the dependence of the reaction rate on the deviation from the equilibrium CO_2_ partial pressure (*n*) was similar for both materials (2.01 for limestone and 2.19 for dolomite), indicating a significant sensitivity of the process to the CO_2_ partial pressure and suggesting that both sorbents require substantial deviation from equilibrium to achieve appreciable reaction rates.

These findings contribute to a better understanding of the kinetic behavior of both sorbents under conditions relevant to CO_2_ capture via chemical looping or gasification with integrated carbon separation, providing key insights for the design and optimization of more efficient and stable systems. The kinetics obtained can be effectively applied to the design and modeling of gasification systems integrated with calcium looping, where moderate calcination temperatures, such as those used here (800–850 °C), enable better thermal integration between the calciner and carbonator, thereby enhancing the overall efficiency and thermochemical feasibility of the process at an industrial scale.

## Figures and Tables

**Table 1:** Physical and textural properties of the sorbents employed.

	Limestone	Dolomite	Half-Calcined Dolomite
Particle size (μm)	250–355	250–355	250–355
Average diameter (μm)	289.5	301.4	304.6
Density (kg/m^3^)	2734.5	2878.4	2315.8
BET surface area (m^2^/g)	0.44	0.05	0.12
Average pore diameter (nm)	20.98	39.03	58.50
S_0_ (m^2^/m^3^)	19.05 × 10^7^	10.38 × 10^7^	6.84 × 10^7^

**Table 2:** Fitting parameters to determine the sorbent deactivation as a cycle function.

Temperature (°C)	$p_{CO_{2}}$ (bar)	Limestone			Dolomite		
		κ	$X_{r}$	R^2^ (%)	κ	$X_{r}$	R^2^ (%)
775	0.05	0.253	0.000	98.75	-	-	-
800	0.05	0.271	0.000	99.62	-	-	-
	0.075	0.282	0.000	99.64	0.272	0.063	99.89
	0.10	0.417	0.040	99.37	-	-	-
825	0.05	0.318	0.022	99.22	0.286	0.077	99.94
	0.075	0.412	0.035	98.98	0.355	0.080	99.02
	0.10	0.637	0.063	99.43	0.358	0.072	99.47
850	0.05	0.532	0.065	99.88	-	-	-
	0.075	0.573	0.060	99.19	0.367	0.077	99.83
	0.10	0.783	0.073	99.20	-	-	-

## Data Availability

The original contributions presented in this study are included in the article. Further inquiries can be directed to the corresponding author.

## Nomenclature

$A_{0}$	Pre-exponential factor (μm/s)
$d_{p}$	Particle diameter (μm)
$E_{a}$	Activation energy (kJ/mol)
$f\left(p_{CO_{2}}\right)$	Pressure dependent term (-)
N	Calcination–carbonation cycle
n	Reaction order (-)
$p_{CO_{2}}$	Partial pressure of CO_2_ in the feed gas (bar)
$p_{CO_{2},eq}$	Equilibrium partial pressure of CO_2_ (bar)
$p_{H_{2}O}$	Partial pressure of H_2_O in the feed gas (bar)
$p_{N_{2}}$	Partial pressure of N_2_ in the feed gas (bar)
R	Ideal gas constant, 8.314 × 10^−3^ kJ/mol/K
T	Temperature (K)
t	Time (s)
X	CaCO_3_ calcined (mol of CaCO_3_/mol of Ca)
$X_{1}$	Conversion at the first cycle
$X_{N}$	Carbonation effective conversion at cycle N
$X_{r}$	Residual conversion
Greek letters	
α	Fraction of CaCO_3_ calcined to CaO with reference to the moles of CaCO coming from former carbonation
κ	Deactivation constant (-)
$\tau_{S}$	Complete calcination conversion time (s)
Abbreviations	
CLG	Chemical looping gasification
CaL	Calcium looping
CaLG	Calcium looping gasification
FB	Fluidized bed
FBG	Fluidized bed gasification of fluidized bed gasifier
SEG	Sorption-enhanced gasification
